# Evaluation of platelet indices as markers of tuberculosis among children in India

**DOI:** 10.1183/23120541.00734-2023

**Published:** 2024-02-26

**Authors:** Nancy Hilda J, Aishwarya Venkataraman, Kannan Thiruvengadam, Brindha B, Karthick M, Subha S, Sarath Balaji, Elilarasi S, Melanie Smuk, Luke Elizabeth Hanna, Andrew J. Prendergast

**Affiliations:** 1 ICMR – National Institute for Research in Tuberculosis, Chennai, India; 2 Blizard Institute, Queen Mary University of London, London, UK; 3 Institute of Child Health, Madras Medical College, Chennai, India; 4 Joint first authors; 5 Joint senior authors

## Abstract

The function of platelets in the pathogenesis of paediatric tuberculosis remains elusive. In this study, we demonstrate that young children with tuberculosis have an elevated platelet count and platelet/lymphocyte ratio (PLR), as well as a decreased mean platelet volume (MPV), highlighting the significance of these parameters in the diagnosis and prognosis of tuberculosis in children.

## Shareable abstract

**Children with tuberculosis have increased platelet count and platelet/lymphocyte ratio along with decreased mean platelet volume, suggesting that these indices may be useful as adjunct tools in diagnosis of paediatric tuberculosis** https://bit.ly/3Ga4AWT

*To the Editor:*

The function of platelets in the pathogenesis of paediatric tuberculosis remains elusive. In this study, we demonstrate that young children with tuberculosis have an elevated platelet count and platelet/lymphocyte ratio (PLR), as well as a decreased mean platelet volume (MPV), highlighting the significance of these parameters in the diagnosis and prognosis of tuberculosis in children.

Platelets are central to haemostasis but also play major roles in the inflammatory response and in infection containment [1]. Thrombocytosis and low MPV have previously been reported in people with infectious and inflammatory diseases such as tuberuclosis [1]. In particular, deep vein thrombosis (DVT) is observed in adults with severe pulmonary tuberculosis [2, 3]. This may be due to thrombocytosis, increased platelet aggregation and other haemostatic disturbances leading to a hypercoagulable state [2, 4]. Nevertheless, even in children with high platelet numbers, DVT is extremely rare. The presence of platelets in human tuberculous granulomas and an increase in platelet-associated gene transcripts during tuberculosis [3] further implicates platelets in its immunopathogenesis. In addition, platelets play important roles in tuberculosis pathogenesis by releasing CCL17/thymus and activation-regulated cytokine, which is involved in chemotaxis of type 2 helper T-cells [5]. Platelet count, MPV, PLR, and changes in platelet structure and function have been investigated as potential surrogate biomarkers for tuberuclosis [3, 6–12]. Changes in MPV can indicate elevated platelet production and activation, reflecting the inflammatory response associated with tuberculosis [6–8]. Similarly, PLR has been reported as a useful prognostic and diagnostic marker in several conditions, including cancer, cardiovascular disease and inflammatory diseases [9, 11, 13], and more recently, changes in PLR have been described in tuberuclosis [12, 13]. Despite multiple reports on platelet numbers and indices in adult tuberculosis, to our knowledge, reports on changes in platelets in paediatric tuberculosis are lacking.

Here, in a longitudinal cohort of children with newly diagnosed tuberculosis disease or tuberculosis infection (TBI), we explored platelet numbers and indices. Our hypothesis was that platelet count, MPV and PLR serve as valuable markers in distinguishing TBI from tuberculosis disease in children.

This analysis leveraged an ongoing study (www.clinicaltrials.gov identifier number NCT05044910) in Chennai, India, assessing the immune responses to *Mycobacterium tuberculosis* in children <5 years of age. The study was approved by the Institutional Ethics Committee of the Indian Council of Medical Research – National Institute for Research in Tuberculosis, and was registered at Clinical Trials Registry India (identifier number CTRI/2020/03/023916) as well as at clinicaltrials.gov. Caregivers provided written informed consent for children to be included in the study. Three groups of children aged 12–60 months with a median (interquartile range) age of 36 (22–46) months, of whom 51% (79 out of 156) were male, were enrolled from the outpatient clinics of four paediatric hospitals in Chennai, India: tuberculin skin test (TST)-negative (tuberculosis-exposed, TST-negative; n=73); TST-positive (tuberculosis-exposed, TST-positive; n=46); and tuberculosis disease (microbiologically confirmed; n=37). All children were HIV-negative and bacille Calmette–Guérin-vaccinated at birth. TST was performed at enrolment, prior to starting antituberculous therapy (ATT) or isoniazid prophylaxis. Children with tuberculosis disease were treatment-naïve and started ATT after their baseline assessment. All tuberculosis-exposed children (both TST-negative and TST-positive) started isoniazid prophylaxis. All children were followed up at weeks 12 and 24. At each visit, 2 mL EDTA blood was drawn by a trained phlebotomist and haematological analytes were measured using a BC5150 Analyser (Mindray Global, China) within 2 h.

STATA version 15.0 (Stata Corporation, College Station, TX, USA) was used for quantitative analysis. A two-sided Kruskal–Wallis test was used to determine statistical significance in all analyses using an α-level of 0.05. At baseline, there were no significant differences between the three groups in age (p=0.19; median age in each group: TST-negative, 36 (22–46) months; TST-positive, 31 (18–46) months; and tuberculosis disease, 29 (17–40) months), sex (p=0.25; males in each group: TST-negative, 44%; TST-positive, 54%; and tuberculosis disease, 59%) or nutritional status (p=0.53; malnourished in each group: TST-negative, 55%; TST-positive, 46%; and tuberculosis disease, 46%). Children were grouped as malnourished based on World Health Organization guidelines (weight for height Z-score between −2 and −3 and/or mid-upper arm circumference between 115 and 125 mm). To examine baseline differences in platelet indices between the three clinical groups, Dunn's test was used with multiple testing correction using Holm's test. We observed significant differences between the groups in platelet numbers (p<0.001), PLR (p=0.0048) and MPV (p<0.001) at baseline (figure 1a). Platelet counts were significantly higher in children with tuberculosis disease (449 (376–524) × 10^3^ cells per μL) compared to TST-positive (370 (307–450) × 10^3^ cells per μL) and TST-negative (345 (281–397) × 10^3^ cells per μL) children. Similarly, PLR was significantly higher in children with tuberculosis disease (81.2 (61.8–105.7)) compared to TST-positive (71.2 (48.8–98.5)) and TST-negative (62.6 (43.4–76.8)) children. In contrast, MPV was significantly lower in children with tuberculosis disease (8.4 (7.7–8.9) fL) compared to TST-positive (8.8 (8.4–9.4) fL) and TST-negative (9.3 (8.4–10.0) fL) children. Over time, platelet count (p=0.002) and PLR (p=0.01) decreased significantly in children with tuberculosis disease. However, by week 12 (for PLR) and week 24 (for platelet count), the three clinical groups had no evidence of difference (figure 1a and 1b). By contrast, the MPV values remained persistently lower in children with tuberculosis disease, even at week 24. In both TST-negative and TST-positive children, the measured platelet indices did not vary significantly through the follow-up period, except for an increase in PLR in TST-negative children by week 24 (p=0.04) (figure 1b). To further examine the change in the indices over time in each group, generalised estimating equations with an independent correlation structure and robust standard errors, including an interaction term (time×group), were used after adjusting for age, sex and nutritional status. This analysis confirmed that both platelet count (p=0.015) and PLR (p=0.005) significantly reduced over time, whereas MPV showed no significant change (p=0.70) during follow-up in children with tuberculosis disease (figure 1c).

**FIGURE 1:**
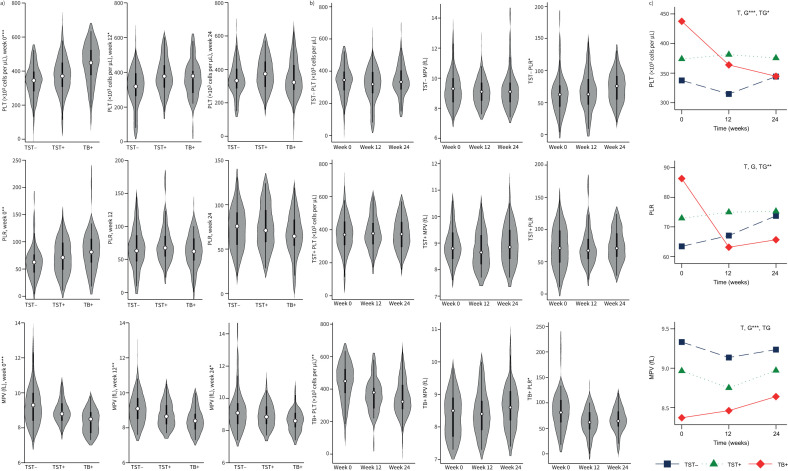
Participant platelet profiles according to tuberculosis (TB) disease and TB infection status. a) Comparison of platelet (PLT), platelet/lymphocyte ratio (PLR) and mean platelet volume (MPV) between the three study groups (TB-exposed, tuberculin skin test (TST)-negative (TST−); TB-exposed, TST-positive (TST+) and TB disease (TB+)) of children aged 12–60 months, at three time-points: baseline (week 0), 12 weeks and 24 weeks. b) PLT, MPV and PLR within each study group throughout follow-up. c) Two-way interaction plots showing the average and trend in the observed value of PLT, PLR and MPV over time-points in each study group. Data are adjusted for age, sex and nutritional status. T: time; G: group; TG: interaction of time and group. *: p<0.05; **: p<0.01; ***: p<0.001.

Since platelets are not only involved in coagulation but also in immune responses, indices of number, size and function may prove useful in terms of assessing immune status and disease progression. Previous studies have reported MPV and PLR to be correlated with inflammation in tuberculosis disease [11, 12, 14]. Likewise, PLR has been reported to be useful in identifying tuberculosis disease in COPD patients [13]. We therefore wanted to extend these findings in children to understand whether platelet number and indices might differ between children with and without tuberculosis disease. Our finding of increased platelet numbers and PLR, together with decreased MPV, in children with tuberculosis disease prior to treatment suggests that these indices may be useful as adjunct tools in identifying whether a child has tuberculosis disease or TBI. The decline in platelet numbers and PLR observed in children with tuberculosis disease following treatment suggests that monitoring these markers might indicate response to therapy over time. However, this observation has to be evaluated in larger cohorts to investigate the potential of these haematological analytes as surrogate indicators for diagnosis and monitoring in tuberculosis disease. However, despite being an inflammatory marker, the consistent MPV levels observed throughout ATT are intriguing. Further research is required to understand the interplay between haematopoiesis, inflammation and immunological responses involving platelets in children receiving ATT.

Our study is limited by having a small sample size and being restricted to children <5 years. We exclusively included children with microbiologically confirmed tuberculosis. Furthermore, we were unable to repeat TST on children who had negative TST results at enrolment. However, this analysis is a part of a larger ongoing study, and our principal aim was to evaluate whether platelet number and indices have potential as adjunctive prognostic or diagnostic tests in children with tuberculosis. Given that diagnosis of tuberculosis in children is often challenging, we suggest that review of available haematological profiles may be helpful as additional investigations and may provide valuable information about the tuberculosis status of the child. Our observations highlight the importance of platelets and associated indices in tuberculosis immunopathogenesis, which warrants further exploration. Our results need to be tested in larger cohorts to understand whether platelets may serve as surrogate tools to differentiate tuberculosis disease from asymptomatic TBI and/or healthy children. Taking this research further, assessing platelet levels and indices throughout ATT in a multicentre study would elucidate the importance of these parameters in the diagnosis and prognosis of tuberculosis in children.

In summary, young children with tuberculosis have an elevated platelet count and PLR, along with a decreased MPV, suggesting that these platelet parameters may be plausible adjunctive tools in the diagnosis of paediatric tuberculosis. The platelet count and PLR decrease over time during ATT, suggesting potential value in monitoring response to treatment.
